# Supplementary material for: Facilitators and barriers for the implementation of a transmural fall-prevention care pathway for older adults in the emergency department
Source: PLoS One. 2024 Dec 31;19(12):e0314855. doi: 10.1371/journal.pone.0314855 (PMC11687785; doi:10.1371/journal.pone.0314855)
Supplement: S2 Appendix — (DOCX) [file pone.0314855.s002.docx]

**Interview Guide Patients**

| **Themes** | **Questions** |
| --- | --- |
| Introduction | Introduction about older adults and falls, ED revisits and the current concept of the TFCP. The aim is to prevent fall-related ED revisits in older adults. Show the current seven step concept to the patient. Introduce the themes which will be discussed. |
| Emergency Department | - How did you experience your visit to the ED? - How did you experience care at the ED? - Are you motivated to participate in actions to prevent another ED visit? - At what moment during your ED visit do you feel was most suiteable to be approached about a TFCP? - **What information would you have liked about the TFCP and in which form? (e.g. verbal, pamphlet, video)?** - **How can we motivate patients at the ED who would need a TFCP to make use of the TFCP?** |
| Fall risk assessment | - How were your first days home after the ED visit? - How did you feel during your first days after the ED visit? - Did a healthcare professional contact you during the first days after your ED visit? (GP, PN or others) - **What are the most important themes in the fall risk assessment during the home visit by a PT?** - Is it important to you that the PTs discus their findings with you and why? |
| Communication between PT and GP | - Do you want your opinion included in the communication from the PT to the GP/PN and why? - Do you want a copy of the letter from the PT to the GP? |
| GP/PN | - Did a healthcare professional contact you during the first days after your ED visit? (GP, PN or others) (Also mentioned before) |
| Interventions | - Do you prefer personalised care above general care programmes? - Are you familiar with fall prevention programmes, did you make us of them in the past? If so, how did you experience it? - **What would withhold you from participating (again)?** - **What would stimulate participation?** - What are important components of a fall prevention program according to you? - What would you need to contine participation in a fall prevention program? - **What could withhold or stimulate others from participating? Wat denkt u dat anderen tegen zou kunnen houden om mee te doen?** - How would insurance of fall preventive care influence participation? |
| Optional | - Extra subjects which could arise during conversation or due to follow-up questions |
