# Supplementary material for: Facilitators and barriers for the implementation of a transmural fall-prevention care pathway for older adults in the emergency department
Source: PLoS One. 2024 Dec 31;19(12):e0314855. doi: 10.1371/journal.pone.0314855 (PMC11687785; doi:10.1371/journal.pone.0314855)
Supplement: S3 Appendix — (DOCX) [file pone.0314855.s003.docx]

**Interview Guide Healthcare professionals, group interview 2**

| **Themes** | **Questions or description** |
| --- | --- |
| 1. Start and introduction (17.00-17.15) | PowerPoint presentation about the TFCP and study. The goal of today is to gather facilitators and barriers for the implementation of the TFCP. Informed consent, ask if there is any objection for recording the interview, start recording if not |
| 1. General   (17.15-17.25) | 1. How do you feel about the concept TFCP? 2. Is it complex? 3. What are you already doing and why? 4. What do you think about when we talk about frail older adults in your care? 5. What do you notice about ED revisits? 6. How do you experience the frailty of an older adult with fall related injury at the ED (question for ED staff) or an older adult with fall related injury who has visited the ED (question for PTs) |
| 1. Emergency department   (17.25-17.45) | Questions for emergency department staff:   - What makes a patient frail? - How can we help facilitate to use the Clinical Frailty score to identify patients for the TFCP? How much time would a CFS assessment take? - What is the current care for patients who visit the ED with fall related injuries? - Do you feel that a TFCP is missing from current practice? - How can we facilitate patient inclusion, who should coordinate this? - Is the ED ready for implementation? - What is present or missing (leadership, resources, knowledge)? - **What are facilitators and barriers for the ED to participate in the TFCP?** - **How can we make participation for the ED as easy as possible?** |
| 1. Communication between ED, PT and GP   (17.45-18.00) | Questions for ED and PTs:   - What is the current communication between ED and primary care PTs? - **What would be best practice according to you?** - How do you feel about Siilo, LSP, C-Boards? - What is the minimal amount of information the ED can sent and the PT wants to receive? - **What are facilitators and barriers in this communication?** - How can we standardise this communication?   - Templates?   Questions for ED:   - What is the current communication between ED and GP/PN? - **What would be best practice according to you?** - What is the minimal amount of information the ED can sent and we feel the GP wants to receive? - **What are facilitators and barriers in this communication?** - How can we standardise this communication?   - Templates? |
| 1. Fall risk assessment   (18.00-18.15) | Questions for primary care PTs:   - **How would you prefer the action after the ED? Should PT call the patient or should the patient call the PT?** - **How did you experience the interRAI assessment in the past? What could be improved?** - **How could we help with the assessment and interpretation of the interRAI to make it as easy as possible?** - **What do you need to aid the shared-decision making process? (time, money)**   Question for everyone:   - How would you construct the medication verification process in the TFCP for dosage, side-effects and interactions? Should the pharmacy be involved |
| 1. Communication between PT and GP   (18.15-18.25) | Question for PTs:   - How do you experience the current communication between PTs, GPs and PNs? - **What would be best practice according to you (Siilo, phone, ZorgMail, LSP, C-boards)?** - What is the minimal amount of information the PT can sent and we feel the GP wants to receive? - **What are facilitators and barriers in this communication?** - Are the interRAI results easily interpretable for the GP and PN? - What feedback does the PT want to receive from the GP? - Who coordinates this? - How can we standardise this communication?   - Templates? |
| 1. Communication between GP and intervention agents   (18.25-18.35) | Questions for PTs   - How do you currently experience referrals from the GP and PN? - What is the minimal amount of information the PT wants to receive? - **What are facilitators and barriers in this communication?** |
| 1. Interventions   (18.35-18.45) | Questions for PTs   - What is the current practice for organising intervention care for older adults with fall related injuries in your region? - Do you feel healthcare professionals are aware of the content of interventions from other healthcare professionals? How could we improve this? - Are interventions accessible? - **What are facilitators and barriers in this section of the TFCP?** |
| 1. Room for new subjects |  |
