# Supplementary material for: Facilitators and barriers for the implementation of a transmural fall-prevention care pathway for older adults in the emergency department
Source: PLoS One. 2024 Dec 31;19(12):e0314855. doi: 10.1371/journal.pone.0314855 (PMC11687785; doi:10.1371/journal.pone.0314855)
Supplement: S4 Appendix — (DOCX) [file pone.0314855.s004.docx]

**Interview Guide Healthcare professionals, group interview 2**

| **Themes** | **Questions or description** |
| --- | --- |
| 1. Start and introduction (13.00-13.10) | PowerPoint presentation about the TFCP and study. The goal of today is to gather facilitators and barriers for the implementation of the TFCP. Informed consent, ask if there is any objection for recording the interview, start recording if not |
| 1. General   (13.10-13.20) | - How do you feel about the concept TFCP? - Is it complex? - How do you feel about the role of the GP and PN in this care pathway? - What do you notice about ED revisits? - How do you experience the frailty of an older adult with fall related injury who has visited the ED? |
| 1. SEH | - |
| 1. Medication verification   (13.20-13.30) | - How would you construct the medication verification process in the TFCP for dosage, side-effects and interactions? Should the pharmacy be involved |
| 1. Communication between ED and GP   (13.30-13.45) | Question for ED and GP:   - How quick do GPs receive the ED discharge letter - Is it complete? - How quickly after receiving the discharge letter is it read? - **What would be best practice for communication between ED, PT and GP according to you?** - What timeframe would be feasible for the GP or PN to contact the patient? - What is the minimal amount of information the ED can sent and we feel the GP wants to receive? - **What are facilitators and barriers in this communication?** - How can we standardise this communication?   - Templates? |
| 1. Fall risk assessment | - |
| 1. GP/PN   (13.45-13.55) | Questions for GP/PN:   - Discussion about receiving the results   - How would you prefer to receive the results (physiotherapists suggested by phone)?   - How do you currently experience communication between physiotherapists and practice nurses/general practitioners?   - What do you see as the best way to arrange this line of communication (phone, secure email)?   - What should this communication at least include?   - What are the facilitating and hindering factors in this communication?   - Are the results from the interRAI and the subsequent recommendations understandable for the practice nurse/general practitioner? - Contact with the patient about results   - What are your thoughts on this? - What could the feedback to the physiotherapist look like? - Contact with the intervention agents |
| 1. General | - What do you regard as your responsibility in the TFCP and what is the responsibility of the ED and PTs? |
| 1. Room for new subjects |  |
