# Supplementary material for: Facilitators and barriers for the implementation of a transmural fall-prevention care pathway for older adults in the emergency department
Source: PLoS One. 2024 Dec 31;19(12):e0314855. doi: 10.1371/journal.pone.0314855 (PMC11687785; doi:10.1371/journal.pone.0314855)
Supplement: S5 Appendix — (PDF) [file pone.0314855.s005.pdf]

Translations and original paragraphs

Q1

Translation presented in the manuscript:

WMC: “Could patients be motivated to participate in indicated care if the GP or PN would contact you after he or she receives the transferal from the therapist?”

P-9: “Absolutely, presuming a good relationship between patient and GP.”

Original paragraph from Dutch transcript:

WMC: Zou het de patiënt nog extra kunnen motiveren als de huisarts dan even contact met hem opneemt om dat door te spreken? Of de praktijk assistent?

P-9: Ja euh, euh, dat zeker? Euh euh. Er vanuitgaande dat er tussen patiënt en huisarts toch een zekere vertrouwensrelatie is. (Transcriptie 009, Pos. 260-262)

Q2

Translation presented in the manuscript:

P-2: “Yes, if I would trust GPs again than it would be okay.”

Original paragraph from Dutch transcript:

WMC: Stel u had wel een goede band gehad met uw huisarts, zou het dan helpen als hij u zou bellen van hé ik heb net van de fysiotherapeut de analyse binnen gekregen?

P-2: Ja dat wel, als ik weer vertrouwen in huisartsen zou hebben zou dat wel oke zijn. (Transcriptie 002, Pos. 158-161)

Q3

Translation presented in the manuscript:

WMC: “How could the physiotherapist stimulate you to do exercises?”

P-8: “If you feel the necessity yourself, then I think you’ll do it, but I don’t have that feeling right now. [Patient 8 got a brace from the hospital] However, when my son said: “If we bike to the garden next week, you’ll wear your brace”. I said: “I will”.

Original paragraph from Dutch transcript:

WMC: Hoe kan die therapeut dan, stel dat die therapeut zegt: het zou goed zijn om oefeningen te gaan doen. Hoe zou hij u kunnen stimuleren om dat te gaan doen?

P-8: Hm bepaalde dingen ja als je zelf het gevoel hebt dat het nodig is dan doe je dat denk ik. Want ik heb nu helemaal niet het gevoel van nou moet ik op eens. Nee maar mijn zoon zegt wel ik heb van het ziekenhuis toen kreeg ik: mam volgende week gaan we wel naar de tuin fietsen dan doe je wel je ding om hoor. (Transcriptie 008, Pos. 235-238)

Q4

Translation presented in the manuscript:

GP-1: “We can’t, with all respect, implement fall prevention if the ED is unaware of the interventions that have already been initiated by the GP.”

Original paragraph from Dutch transcript:

GP-1: We kunnen niet zeg maar vind ik hé met met met alle respect, proberen valpreventie te voorkomen als we niet kunnen inzien dat er al dingen zijn gedaan van te voren.

(Transcript Huisartsen/POH, Pos. 59)

Q5

Translation presented in the manuscript:

WMC: “What are factors that could improve or hinder the communication between you (GP’s office) and the hospital?”

PN-1: “I would like a contact person in the hospital for me as PN, and a call so we could discuss the discharge letter.”

Original paragraph from Dutch transcript:

WMC: ja, wat ik nog even nieuws was nog even één stap terug weer naar de communicatie, van de twee naar de eerste lijn. Wat zijn nou factoren die die communicatie juist kunnen bevorderen of juist kunnen belemmeren waarin, wat jullie tot nu toe ook ervaren hebben als jullie communicatie hebben gehad met het ziekenhuis?

PN-1: Nee, ik zou heel graag een contactpersoon willen in het ziekenhuis als POH. die daar over gaat. Eh .Ja, ik zou eigenlijk. Ja, ook heel graag toch ook even een Belletje willen? Eigenlijk even. Ja, een Belletje. En. Om het door te bespreken en. De ontslagbrief. Zeg maar, ik merk dat de ontslagbrief. Vaak later. Is uit het ziekenhuis dan! Hoe zeg je dat nou? Ja, dat ie dat ie vaak wat later Kom dan is de patient En meestal al.Thuis, ik zou ik zou. Ja. graag Meerdere vlak. (Transcript Huisartsen/POH, Pos. 137-138)

Q6

Translation presented in the manuscript:

EDN-1: “For the future I believe a web based programme like Point is more suitable. Point is currently used by our transfer nurses to organise post hospital care. I think that will be easier.”

MvB: “And how does something like Point work?”

EDN-1: “Right now we work with an ambulance planner to order transport, it is sort of the future. If we can simply go to an address, indicate with a few clicks who and what is coming,

like a physiotherapist, I think that works the fastest and will be the best plan in the long term.”

<u>Original paragraph from Dutch transcript:</u>

EDN-1: Ja, denk die groep waar ik het over had, ja dan de ander, maar ik ik, ik denk dat dit vooral iets is voor een onderzoeksfase en ik zou voor een permanent iets toch veel meer denken aan een web based, programma's zoals we gebruiken nu point dat is dan ook een web baseds programma waarin de transfer pleegkundigen klinische patiënten nazorg gaat organiseren. Dat dat Ja, dat ik denk dat dat veel makkelijker werkt en dus word alles...

MvB: En hoe werk zo iets?

EDN-1: Nou, ja, dan kan je zelf inrichten we hebben steeds meer web based programma's zoals we werken nu ook met een ambulance planner waar we vervoer bestellen, gewoon op een webbased manier, en dat wordt een beetje manier van werken van van de toekomst. Dus dat wordt eigenlijk Normaal op heel veel vlakken ja, als wij gewoon naar een adres kunnen gaan, daar met een paar klikken kunnen aangeven wie en wat er komt, en zo'n Fysio gaat naar Hetzelfde adres. Dat werkt het snelst denk ik, dus ik denk dat over langere-termijn het beste plan zal zijn.

MvB: Hoe weet je dan welke fysio je moet klikken?

EDN-1: Nou ja, dat dat zijn dingen waar je natuurlijk over na moet gaan denken. Dat zijn dat moet ontworpen worden, dus daarom zeg ik dat is iets voor lange termijn. (Transcript SEH/FT, Pos. 178-182)

Q7

<u>Translation presented in the manuscript:</u>

P-4: “I also think that if you are in such a situation, you probably also need home care, so from multiple domains healthcare professionals will be approaching you. So that might be a lot of the same from different directions, that does not seem effective to me. […] That is of course a waste of energy and money.”

<u>Original paragraph from Dutch transcript:</u>

P-4: Ja en ik zit ook te denken dat als je in zo’n situatie zit ja dan heb je waarschijnlijk als je alleenstaand bent ook thuiszorg nodig ofso dus dan krijg je ook al vanuit die hoek al die uh ja dan gaan ze ook kijken hoe dus dan komt er wel van veel alles bij elkaar. Als het over te veel schijven gaat lopen dat lijkt mij ook niet handig.

IN: Nee dus dan moeten wij nog eens kijken naar thuiszorg

P-4: Ja dat het niet allemaal van die verschillende uh

WMC: Ja dat niet de praktijkondersteuner met zo’n vragenlijst komt en dan de fysiotherapeut ook nog.

P-4: Ja en hoe stemmen ze dat dan af en wat is er dan al afgestemd en komt dat in een la of uh.

WMC: Of doen ze het allemaal los van elkaar

P-4: Ja en dat is natuurlijk zonde van de energie en geld wat er in gestopt wordt. Ja dus dan ja. (Transcriptie 004, Pos. 136-149)

Q8

Translation presented in the manuscript:

EDN-1: “At the ED, we like practical agreements, so the shorter, the more concise, the clearer, the better.”

Original paragraph from Dutch transcript:

EDN-1: Maar wij houden op de SEH meer van die praktische afspraken, van praktische dingen, omdat er lopen met 20000 onderzoeken op de Spoed en hoe strakker hoe korter, hoe duidelijke, des te beter. (Transcript SEH/FT, Pos. 52)

Q9

Translation presented in the manuscript:

EDN-1: “However, if it is truly about informing about the study and informed consent procedure than I wonder if that is the task of the ED nurse. Screening and selecting for the care, sure.”

Original paragraph from Dutch transcript:

EDN-1: Maar als echt voorlichting is en in inlichten over onderzoek, dan vraag ik me af of dat de taak van de SEH verpleegkundige is. Kijk, screenen en en selecteren prima. (Transcript SEH/FT, Pos. 77)

Q10

Translation presented in the manuscript:

EDD: “We should try to create a feeling that despite hectic times at the ED, the patient could deteriorate over time if we don’t start the TFCP.”

Original paragraph from Dutch transcript:

EDD: De laatste tijd zien we dat één of ander iets. Veels te vaak heb ik het gevoel, dus dat is ook een heel kwetsbare groep. Uh Wat doe je dan? Ja, vind ik heel moeilijke. Trouwens nou, intoxicatie inderdaad, meest of laatst had ik iemand die ook al 20 jaar sterk opiaat gebruikt. Er komt zo naar ons, wat is de kwetsbaarheid van iemand? Zo is dat eenmalig, is al wel 20 keer daarvoor ook gebeurd. Dus inderdaad, bepaalde zegt, dat is heel complex. Iets is niet en dan moet je een gevoel hebben. Interesse hebben dat jij wel bewust, omdat soms door de hectiek heb je daar de tijd ook niet tot de Spoed moet je gevoel, creëren dat dat dat toch iemand kan, slechter, worden zeg maar als je naar huis gaat zonder enige plan wat we nu proberen te maken. (Transcript SEH/FT, Pos. 123)

Q11

Translation presented in the manuscript:

EDN-1: “My preference is that we simply implement this in our electronic patient record in which we already screen for falls and fall risk. If we could link these questions to a reminder of the TFCP, you can’t really ignore it. But then it has to be built in, that is where we will face other challenges.”

Original paragraph from Dutch transcript:

EDN-1: Mijn voorkeur heeft dat we dat we dat gewoon implementeren in epic waar wij ook gewoon screenen op val, twee vragen hebben en de of waar schat wij in dat iemand val gevaarlijk is. Dus dat ik wel interessante vragen hebben. Moeten we die later ook niet gaan includeren niet nu, maar later misschien wel, en dat daar dat we deze vragen daaraan ophangen en dat dat gewoon uitklapt in epic ja, dan dan kan je d'r eigenlijk niet omheen en dan heb je dat ook zo geregistreerd. Maar dan moet het ingebouwd worden. Dus daar komen we weer met andere uitdagingen te maken.

WMC: Wat denk je van haalbaarheid? Op wat voor termijn zou dat, denk je?

EDN-1: Nou ja, dan moet ik even met epic overleggen met de mensen die daar betrokken zijn, maar dat zou, dat moet wel kunnen, zeker.

WMC: Oké

EDD: Ja, maar epic, dat is heel lang, dan moet je niet verwachten. (Transcript SEH/FT, Pos. 90-94)

Q12

<u>Translation presented in the manuscript:</u>

P-6: “I think that participating in the indicated care is the first phase. The next phase is when patients continue with each other. … I think that a patient who needs to lose weight or who is unstable could benefit from behavioural change, not just a short-term intervention.”

<u>Original paragraph from Dutch transcript:</u>

P-6: En die, euh, die hé, die hebben dat gewoon gewoon plezier in dus die. Die uhm, ik denk dat als je die revalidatie doet dat dat de eerste fase is dat ze die revalidatie proces afmaken.

Maar ik denk dat het vervolg proces dat kan ook weer voor de fysio interessant zijn. Het vervolg proces is dat je mensen met elkaar door blijven gaan. Ja, daar zit een voor zit voor dat de fysiotherapeut die eraan meedoet en die een fitnessruimte heeft. Dan zit daar een win in. Want je zou zeggen oooh ik kan die met aan mij binden. Daar ga ik met het een groepje ga ik verder. Ga ik doe ik gewoon een keer in de week. Blijven we dit voor doen. Ja dus ik denk dat. Ik denk namelijk dat mensen die euh of willen af moeten afvallen of instabiel zijn dat die er bij gebaat zijn dat er een soort gedrag verandering ontstaat. Ja en niets dat het op korte termijn eventjes een trainingetje is. (Transcriptie 006, Pos. 321)

Q13

<u>Translation presented in the manuscript:</u>

WMC: “Does it matter if the TFCP is covered by insurance?”

P-5: “I believe it does. … I just think it is bad of them (insurance companies), truly. You can include that too. I just think that if they want people to remain mobile, then physical therapy should receive more attention, especially for older adults because many can’t afford that.”

<u>Original paragraph from Dutch transcript:</u>

WMC: Maakt het nog uit of de zorg verzekerd zou zijn?

P-5: Denk het wel, zeker. Ik vind het gewoon slecht van ze hoor. Dat mogen jullie ook meenemen. Ik vind gewoon dat als ze mensen mobiel willen laten zijn dan moet fysiotherapie net daar grotere aandacht voor uh zeker voor ouderen.

IN: En als zo’n valpreventieprogramma geen fysiotherapie is maar echt een programma apart.

P-5: Ja maar heel veel mensen kunnen dat niet betalen.

(Transcriptie 005. Pos. 345-376)

Q14

Translation presented in the manuscript:

EDN-1: “Patient recognition and inclusion at the ED has to be quick and effective. It should not cost too much time; otherwise, people won’t do it.”

MVB: “Within what time frame should it be possible?”

EDN-1: “Within a few minutes seems right to me”

EDN-2: “Yes, a maximum of five minutes.”

Original paragraph from Dutch transcript:

EDN-1: Dan moet je over nadenken. Ja, dat moet gewoon snel en effectief zijn. Nou effectief, dan kun je dat misschien niet meten maar dat moet niet te veel tijd kosten. Ja, oké, want dan gaan ze mensen dat niet doen.

MVB: En wat is Max tijd binnen? Wat voor tijd moet het kunnen?

EDN-1: Nou, binnen een paar minuutjes moet je iets kunnen invullen lijkt mij!

EDN-2: Ja, vijf minuten.

(Transcript SEH/FT, Pos. 159-163)

Q15

Translation presented in the manuscript:

WMC: “Would it help if the GP would contact you after receiving the transferral from the physiotherapists?”

P-2: “Yes, but you also don’t want to burden doctors, especially during the pandemic. I often interact more with the PN than the GPs themselves.”

<u>Original paragraph from Dutch transcript:</u>

WMC: Stel u had wel een goede band gehad met uw huisarts, zou het dan helpen als hij u zou bellen van hé ik heb net van de fysiotherapeut de analyse binnen gekregen?

P-2: Ja maar ja je wil huisartsen ook niet belasten hoor in de coronatijd. Dit zou dan voor daarna zijn. Ik heb vaak meer met de huisarts-assistent dan met de huisarts zelf. (Transcriptie 002, Pos. 158-171)

Q16

<u>Translation presented in the manuscript:</u>

WMC: “Did you feel an urge for action to prevent another fall while you were at the ED?”

P-4: “No, it was just a dumb accident. … I would like to write the municipality to fix all those uneven paving stones.”

<u>Original paragraph from Dutch transcript:</u>

WMC: Maar u dacht niet, ik merk dat het wat moeilijker gaat met mijn enkels, misschien moet ik daar hulp voor zoeken om te voorkomen dat ik nog een keer val?

P-4: Nee, nee, nee. Ik zou de gemeente aan willen schrijven van doe eens wat met die ongelijke stoeptegels die allemaal verzakken ja. (Transcriptie 004, Pos. 111-113)

Q17

Translation presented in the manuscript:

P-10: “I don’t think it will help, it will solve itself eventually. I will just hold on tight (laughs), I don’t believe it is necessary.”

Original paragraph from Dutch transcript:

P-10: Ja ik denk dat zal wel niet zo helpen, het zal wel loslopen, ik hou me wel vast (lacht). Ik denk dat het niet nodig is. (Transcriptie 010, Pos. 373-374)

Q18

Translation presented in the manuscript:

P-3: “I think I have given up, I have accepted it. I think the same as the doctors says: “Think about your age”. Then I think, I don’t know if I can make progress.”

WMC: “Why do you feel that you can’t make progress?”

P-3: “I feel like a wreck because walking is difficult and I’m always tired. I don’t feel like I can make progress, at my age. […] I think I gave up and that will stay until I die, and I have accepted that even though it is hard. I’m not going to participate in different activities like walking in the woods. I have become lazy and inactive, and I just accepted it.”

Original paragraph from Dutch transcript:

WMC: En waarom bent u dan niet gegaan? Wat? Wat zijn gedachten die u heeft? Waardoor u dacht nou ik, ik ga nog niet.

P-3: Ik denk dat ik hebben opgegeven. Ik heb me daar bij neergelegd dat de. Ik denk ook dat zelfde als wat de dokters zeggen. Denk aan die leeftijd. En dan denk ik ik weet niet of ik dat vooruit kan laten gaan. (Transcriptie 003, Pos. 224-227)

WMC: En. Hoe komt dat dat u denkt dat er niet meer dat er geen winst meer te halen is?

P-3: Ik voel me een wrak omdat die zo moeilijk lopen en dat ik duizelig bent te vaak of te makkelijk en uhm, en dat ik altijd moe ben. Ik heb een beetje ja. Ik laat me zelf over aan televisie en hand werken. En euh ja. En ik e. Ik zie niet in dat ik vooruitgaan. Ja op de leeftijd. (Transcriptie 003, Pos. 234-237)

Q19

Translation presented in the manuscript:

P-3: "I don't want to use a cane for the rest of my life. … I would also like to do it to stop my partner from having to act as caregiver."

Original paragraph from Dutch transcript:

P-3: Ik wil niet uhm, mijn hele leven met een wandelstok. Mijn hele leven, dat klinkt zo raar. Ik ben heel bewust van mijn leeftijd. Ik zou er ook willen doen dat (partner) niet zo als een mantelzorger moet fungeren. (Transcriptie 003, Pos. 549)

Q20

Translation presented in the manuscript:

P-5: "You want to grow old pleasantly don't you? If you become dependent, that is horrible, you want to postpone that as long as possible."

Original paragraph from Dutch transcript:

P-5: Uh ja gewoon toch uh, goh ja toch gewoon zeggen van ja je wil toch wel op een leuke manier oud worden. Toch ja, ik bedoel als je dadelijk afhankelijk wordt dat is toch vreselijk, dat wil je zo lang mogelijk uitstellen. (Transcriptie 005, Pos. 196)

Q21

Translation presented in the manuscript:

WMC: “Would you like for the GP to contact you after the fall risk assessment, to discuss the next steps?”

P-5: “Yes, but I do mind that the GP will have extra work.”

WMC: “Would the contact encourage you to actually take the next steps?”

P-5: “For sure, I think patients would be more motivated if the GP emphasises that they should do it and that interventions will be beneficial for them.”

Original paragraph from Dutch transcript:

WMC: Zou u het fijn vinden als de huisarts, nadat hij dit (resultaten screening) ontvangen heeft, contact met u zou opnemen over de vervolgstappen?

P-5: Ja, ik vind het wel een beetje erg vervelend voor haar dat ze extra werk krijgt. Maar ik denk toch..

WMC: Maar daarmee voorkomt ze ook extra werk in de toekomst.

IN: Ja zo moet je dat maar zien hè.

WMC: Zou dat u ook stimuleren om juist die vervolgstappen te gaan uitvoeren?

P-5: Zeker voor het grote geheel denk ik dat ze veel meer gemotiveerd zijn als de huisarts nog eens zegt van ik zou het maar doen, dat is goed voor je. (Transcriptie 005, Pos. 255-266)

Q22

Translation presented in the manuscript:

P-5: “I want to stay for a while… I know some older adults who have a little ache and therefore remain in their chairs. That is the beginning of the end, you have to stay active, it is that simple.”

Original paragraph from Dutch transcript:

WMC: En waarom wil u mobiel blijven?

P-5: Omdat ik uh nog een tijdje door wil en ik wil niet zoals ik soms mensen al achter de rollator zie lopen. Ik weet ook van mensen die ouder zijn en die hebben even een pijntje hier en een pijntje daar en die komen niet meer uit hun stoel. Dan denk ik van ja dat is het begin van het einde, je moet gewoon blijven bewegen zo simpel is. (Transcriptie 005, Pos. 81-84)
