# Supplementary material for: Facilitators and barriers for the implementation of a transmural fall-prevention care pathway for older adults in the emergency department
Source: PLoS One. 2024 Dec 31;19(12):e0314855. doi: 10.1371/journal.pone.0314855 (PMC11687785; doi:10.1371/journal.pone.0314855)
Supplement: S1 Table — (PDF) [file pone.0314855.s006.pdf]

	001	002	003	004	005	006	007	008	009	010
**Age category**	60-70	70-80	80-90	70-80	70-80	70-80	90-100	80-90	70-80	80-90
**Sex**	Female	Female	Female	Female	Female	Male	Female	Female	Male	Female
**Falls past month**	0	0	2	0	0	0	0	0	0	0
**Fallen past year**	No	Yes	Yes	No	No	No	No	No	No	No
**Living situation**	Alone	Alone	Together	Alone	Alone	Together	Alone	Alone	Alone	Alone
**In-home care**	Yes	No	No	No	No	No	Yes	No	No	Yes
**Familiar with fall prevention**	No	No	No	No	No	No	No	No	No	No
**Days since recent ED visit**	44	47	22	44	51	46	49	45	59	47
**Clinical Frailty Score**	2	3	5	3	2	2	4	3	2	5
**ED Return within 6 months***	No	No	Yes, twice	No	No	No	No	No	Yes, once	Yes, once

*Only the returns to the ED of Amsterdam UMC, location VUmc were collected.
