# Supplementary material for: Facilitators and barriers for the implementation of a transmural fall-prevention care pathway for older adults in the emergency department
Source: PLoS One. 2024 Dec 31;19(12):e0314855. doi: 10.1371/journal.pone.0314855 (PMC11687785; doi:10.1371/journal.pone.0314855)
Supplement: S2 Table — (PDF) [file pone.0314855.s007.pdf]

	001	002	003	004	005	006
**Age category**	40-50	30-40	60-70	20-30	30-40	30-40
**Sex**	Female	Female	Female	Female	Female	Female
**Profession**	Physiotherapists	Physiotherapist	Physiotherapist	Physiotherapist	ED nurse	ED nurse
**Experience in care with older adults, years**	20	10	40	6	12	15

	007	008	009	010	011	012	013
**Age category**	40-50	50-60	60-70	50-60	60-70	30-40	60-70
**Sex**	Male	Male	Female	Female	Female	Female	Male
**Profession**	ED nurse	ED doctor	Practice nurse	Practice nurse	Practice nurse	Practice nurse	GP
**Experience in care with older adults, years**	10	15	41	30	43	22	30
