# Supplementary material for: Facilitators and barriers for the implementation of a transmural fall-prevention care pathway for older adults in the emergency department
Source: PLoS One. 2024 Dec 31;19(12):e0314855. doi: 10.1371/journal.pone.0314855 (PMC11687785; doi:10.1371/journal.pone.0314855)
Supplement: Attachment — Submitted filename: Response to Reviewers.docx [file pone.0314855.s009.docx]

Dear Editor,

We would like to thank you for considering our manuscript “Facilitators and barriers for the implementation of a transmural fall-prevention care pathway for older adults in the emergency department” for publication in PLOS ONE. We are grateful for the constructive feedback we have received from the reviewers. We have considered the revisions and made adjustments in the manuscript based on the reviewers’ suggestions. Please see below our responses to the reviewers’ questions and comments on a point by point basis.

Kind regards, on behalf of all co-authors,

Wilmar Charmant, MSc

**General comments**

1. Kindly modify your abstract by highlighting key specific findings or insights from the themes and sub-themes from your study. In addition, "the conclusion could emphasize the potential impact of these findings on fall-prevention strategies in emergency departments.".

**Authors response:** We have specified the key themes and the specific finding in the result section of the abstract (Line 37-47). Additionally, we have added the potential impact to the abstract’s conclusion (Line 51-52).

2.  In your Methods section kindly justify your sample size selection, including inclusion and exclusion criteria. And also elaborate a bit further on the demographic characteristics of your study participants. Kindly elaborate on your qualitative methods including who conducted the interviews, pilot testing, and why you chose individual interviews/ focus groups and who participated in or conducted the focus group discussions, and who transcribed the data, etc.

**Authors response:** We added the description of sample size, inclusion- and exclusion criteria into “Participant recruitment and sampling” (Line 109-128). Demographic characteristics of participants are described in the “Results” section (Line 225-237). In “Data collection”, we describe who conducted the interviews/focus groups, pilot testing and who transcribed the data (Line 160-184).

3. Kindly submit a copy of your Interview guide as a supplementary file to accompany your revised manuscript.

**Authors response:** We have added the interview guides to the supplementary files (S2, S3 and S4).

4. In your results section kindly add more quotations where necessary to better illustrate your findings. Kindly reorganize your themes and sub-themes for better clarity and depth. Kindly clarify confusing definitions or statements such as 'the shared decision-making conversation'  and "implementation strategies". Also clarify 'barriers' and 'facilitators' in your study.

**Authors response:** We have added more quotations in the results in sections that the reviewers indicated (Lines 344-347, 360-365, 385-388, 421-422 and 440-443). Moreover, we have defined implementation strategies in the background section (Lines 82-83) and clarified “shared decision-making” by removing “conversation” and adding a reference (Line 150-151). At the start of the results section we have added a sentence clarifying why factors are portrayed as either facilitators or barriers (Line 248-250). We have discussed reorganisation of the themes, especially 2b) fall risk assessment content. While respecting the reviewer’s suggestion, we believe that the theme should stand on its own as it discusses a standalone part of the TFCP. We did change the title of 2b to fall risk assessment domains, similar to the world guidelines for fall prevention and management for older adults.

4. "The discussion could benefit from a more extensive comparison with existing literature, discussing how these findings align or differ from previous studies". Consider including 'a discussion on the use of a constructivist paradigm in this study.' Also, include a clear of statement of Strengths and Limitations of your study after the Discussion section.

**Authors response:** We have extended our discussion with a more in depth continuation on interprofessional collaboration, high staff turnover in the ED, and the reality of implementation (Line 731-754). Additionally, we included a paragraph on the influence of a constructivist paradigm and the lack of framework in the development of the interview guide (Line 777-799). A paragraph of Strengths and Limitations is included after the Discussion (Line 755-799).

5. Since this is a qualitative study, please submit a fully completed COREQ or SRQR Checklist as a supplementary file with your revised manuscript. And in your Methods include a statement that the study was completed in accordance with either of these guidelines.

**Authors response:** In S1, a fully completed COREQ checklist is present. It is also mentioned in the first paragraph of Methods (Line 96-97).

6. Kindly address all other issues raised and annotated by Reviewer 2, including providing appropriate citations where necessary.

Journal Requirements:

When submitting your revision, we need you to address these additional requirements.

1. Please ensure that your manuscript meets PLOS ONE's style requirements, including those for file naming. The PLOS ONE style templates can be found at 
<https://journals.plos.org/plosone/s/file?id=wjVg/PLOSOne_formatting_sample_main_body.pdf> and 
<https://journals.plos.org/plosone/s/file?id=ba62/PLOSOne_formatting_sample_title_authors_affiliations.pdf>

2. PLOS requires an ORCID iD for the corresponding author in Editorial Manager on papers submitted after December 6th, 2016. Please ensure that you have an ORCID iD and that it is validated in Editorial Manager. To do this, go to ‘Update my Information’ (in the upper left-hand corner of the main menu), and click on the Fetch/Validate link next to the ORCID field. This will take you to the ORCID site and allow you to create a new iD or authenticate a pre-existing iD in Editorial Manager. Please see the following video for instructions on linking an ORCID iD to your Editorial Manager account: <https://www.youtube.com/watch?v=_xcclfuvtxQ>

**Authors response:** Wilmar Charmant’s ORCID ID is registered in the Editorial Manager

**Reviewer 1 comments to the manuscript:** Facilitators and barriers for the implementation of a transmural fall-prevention care pathway for older adults in the emergency department.

**Manuscript** #: PONE-D-23-33190

**Title:** Facilitators and barriers for the implementation of a transmural fall-prevention care pathway for older adults in the emergency department

**Authors**: Willem Maarten Charmant; Barbara Snoeker; Hein van Hout; Ilse Nauta; Femke Boonstra; Edwin Geleijn; Cindy Veenhof; Prabath Nanayakkara

**Article type:** Research Article

| Comments | Possible changes | Line | Author’s response | Adjusted lines in the unmarked version |
| --- | --- | --- | --- | --- |
| It was a pleasure reviewing your work. Overall, the paper seems well-structured and informative, with a clear focus on a significant healthcare issue.  However, would benefit from some minor edits. See below |  |  | Thank you for your compliments regarding our work. |  |
| The abstract provides a clear overview of the study, its methods, and the main findings. It succinctly encapsulates the study's purpose, methodology, and conclusions.  While your abstract mentions the main themes, it could benefit from briefly highlighting key specific findings or insights from these themes. Also, the conclusion could emphasize the potential impact of these findings on fall-prevention strategies in emergency departments. |  | Abstract | We have specified the key themes and the specific finding in the result section of the abstract. Additionally, we have added the potential impact on EDs and primary care to the abstract’s conclusion. | (Line 37-47).  (Line 51-52). |
| Concerning your sample size justification, while the number of participants is mentioned, the rationale behind choosing ten older adults and thirteen healthcare professionals could be elaborated for clarity. Also, more information on the diversity of the participant group (e.g., demographics, professional backgrounds) could enhance the understanding of the study's applicability. |  | Methods | We based our sample size on data saturation. Therefore, sample size was not determined prior to the interviews. Even though, after seven patient interviews, we felt that we achieved data saturation we continued up to ten interviews. We did this to make sure we achieved data saturation. For the healthcare professionals, we based our sample on the inclusion of representatives from each domain: ED, physiotherapy and GPs.  The demographics of the participants are included in Table 2 in the Results. | Line 202-204 |
| Kudos on this section. The results were well-organized, providing a structured overview of findings. However, more integration of direct quotes or specific examples from the interviews and focus groups could enrich the narrative and support the findings. |  | Results | Thank you for your compliments. We have added more quotes to the results section. | (Lines 344-347, 360-365, 385-388, 421-422 and 440-443) |
| The discussion could benefit from a more extensive comparison with existing literature, discussing how these findings align or differ from previous studies.  What were the limitations in your study. |  | Discussion | We have elaborated our discussion with a more in depth continuation on interprofessional collaboration, high staff turnover in the ED, and the reality of implementation. Additionally, we included a paragraph on the influence of a constructivist paradigm and the lack of framework in the development of the interview guide. A paragraph of Strengths and Limitations is found after the Discussion. Our main limitations are described in strengths and limitations. | (Line 731-754).  (Line 777-799). |

**Reviewer 2 comments to the manuscript:** Facilitators and barriers for the implementation of a transmural fall-prevention care pathway for older adults in the emergency department.

**Manuscript** #: PONE-D-23-33190

**Title:** Facilitators and barriers for the implementation of a transmural fall-prevention care pathway for older adults in the emergency department

**Authors**: Willem Maarten Charmant; Barbara Snoeker; Hein van Hout; Ilse Nauta; Femke Boonstra; Edwin Geleijn; Cindy Veenhof; Prabath Nanayakkara

**Article type:** Research Article

| Comments | Possible changes | Line | Author’s response | Adjusted lines |
| --- | --- | --- | --- | --- |
| Thank you for the opportunity to review this manuscript. It focuses on a highly clinically relevant issue, namely, fall patients. In terms of the manuscript's quality regarding qualitative studies, several revisions are needed. See comments below. |  |  | Thank you for your compliments regarding our study. |  |
| On line 81, it is written, 'to develop implementation strategies,' but often, we choose existing described strategies, as seen in, for example, Powell et al., 2012, 2015, rather than developing our own implementation strategies. | So, perhaps it is more accurate to write 'to select and/or develop...' | Line 81 | Adjusted to “select and develop”.  Waltz TJ, Powell BJ, Fernández ME, Abadie B, Damschroder LJ. Choosing implementation strategies to address contextual barriers: diversity in recommendations and future directions. Implement Sci. 2019;14(1):42. | Line 83-86 |
| The section on the epistemological standpoint lacks proper references. |  | Line 97-101 | Reference 21 described the epistemology “radical subjectivism”. For further clarification, we now added the paper by Mark Olssen (REF 26) on the epistemology of constructivism. This does not use the same term “radical subjectivism” but describes the same concept. | Line 100-106 |
| In line 121 is written ‘The healthcare professionals were  122 familiar with care for older adults after a fall’. What does familiar means in the context of recruitment of health care professionals?  Were other selected inclusion criteria used, such as general professional experience and experience with the specialty? Why or why not?" |  | Line 121-122 | Familiar means that the healthcare professionals regularly treated older adults within their profession. We changed the sentence for more clarification.  Physio- and occupational therapists were experienced with fall risk assessments as they were affiliated with the pilot study. We have added thist to “Participant recruitment and sampling” | Line 115-116  Line 116-118 |
| Could you elaborate a bit more on the choices you made regarding settings? Why were interviews conducted in the patients' homes and not at the hospital? Why did you choose to categorize healthcare professionals into primary and secondary staff when your objective was to develop a cross-sectoral pathway? |  | Line 126-133 | Patient interviews were conducted at their home so they would be more comfortable. Furthermore, by offering to come to them, patients would be more likely to participate as they would not have to travel themselves. We described our choices more detailed now in Setting.  We tried to get all healthcare professionals into one focus group but logistical barriers due to the COVID pandemic made this impossible. Therefore we divided it into two groups. This is a limitation of the study and is described in the paragraph strengths and limitations of the discussion section. | Line 133-134  Line 761-768 |
| On line 136, it is written ‘We designed the concept of TFCP for older adults after a fall-related ED visit’. I wonder who 'we' are. Is it the research group behind the study? What is the basis for developing this program? Is it based on experiences, evidence-based literature, inputs from users etc.? How was this intervention developed?" |  | Line 236-145 | The concept TFCP was designed by the research group based on prior experiences from the pilot study. In the pilot study they designed the care pathway together with a consortium of healthcare professionals and patient representatives. Based on lessons learned, the new concept TFCP was designed, together with the same consortium.  Hepkema BW, Köster L, Geleijn E, E VDE, Tahir L, Osté J, et al. Feasibility of a new multifactorial fall prevention assessment and personalized intervention among older people recently discharged from the emergency department. PLoS One. 2022;17(6):e0268682.  To clarify, we have added this to the manuscript in “The transmural fall-prevention care pathway”. | Line 144-145 |
| In line 152 it is mentioned ‘were asked for their demographic characteristics’. It's a very broad and generalized statement. Could you elaborate on which demographic data and why these? |  | Line 152 | We added a new sentence which describes the specific demographic characteristics. These were chosen to give the reader a general impression of the participants. The more elaborate demographics in the appendix can be used to match the characteristics with quoted participants. | Line 161-168 |
| Line 155 was the interview guide develop in collaboration with the entire research group or selected researchers. Who and why? |  | Line 155 | The interview guide was developed with the research group and consortium to ensure we gathered the facilitators and barriers on multiple domains.  We have added this to the text. | Line 169-171 |
| On line 156 it is written ‘After and during the discussion of  157 these themes, we reserved time for new input’. What does ‘we reserved time for new input’ means? Who was supposed to provide new inputs? When? Was it part of a pilot test of your interview guide or...? |  | Line 156 | This meant that we reserved time for participants to bring up new subjects that we did not define prior. This was already a part of the pilot interview guide. We adjusted the sentence for clarification. | Line 171-172 |
| Why are WMC and IN conducting individual interviews? Who are they? Similarly, why are WMC, FB, MvB, and EG conducting the focus group interviews? Did all four participate in each focus group interview, or were they divided? How many patients in total participated, and how was this number chosen? The same goes for healthcare professionals? |  | Line 159-162 | WMC and IN conducted the individual interviews as they had the expertise and availability to conduct the interviews. IN was a student intern. WMC was present in all interviews. IN in most of them.  The abbreviations are based on the author initials.  WMC is W.M. Charmant  IN is Ilse Nauta  We described who they are in “Personal characteristics of the interviewers”  WMC, FB, MvB and EG were present in both focus groups.  10 patients participated in total. The number was not picked prior to the study but based on data saturation. We believed we achieved data saturation after 7 interviews but continued up to ten interviews to be sure.  We tried to have multiple healthcare professionals from the same profession/working place e.g. the ED (4), primary care physiotherapy (4) or general practitioner offices (5). | Line 179-195 |
| "Why did you choose focus group interviews instead of group interviews? What was your purpose with the focus group interviews? Which references did you use regarding interviews and focus group interviews? |  |  | Focus groups generally allow for more interaction than (group) interviews as the focus group leader plays the role of discussion facilitator. In group interviews the researcher would be more of an interviewer in which a list of questions is used with little room for discussion.  We wanted to see the direct reaction of healthcare professionals on each other’s visions on facilitators and barriers. Especially the facilitators and barriers that are interprofessional.  Leung FH, Savithiri R. Spotlight on focus groups. Can Fam Physician. 2009 Feb;55(2):218-9. PMID: 19221085; PMCID: PMC2642503.  Jackson P. Focus group interviews as a methodology. Nurse Res. 1998 Oct 1;6(1):72-84. doi: 10.7748/nr.6.1.72.s7. PMID: 27702166.  We have added this to ”Setting”. | Line 139-141 |
| Was the interview guide outlined in the table used for individual interviews with the patients? It seems that several of the themes would be challenging for a patient to respond to. What did the interview guide look like for the patients? |  | Table 1 | The interview guide for patients consisted roughly of the second column. The interview guide for the focus groups was the third column of the table. Elaborate interview guides have been added to the appendices.  If patients had no experience with one of the topics, they were asked to imagine how they would experience it. | S2, S3, S4 |
| In line 165, it is written: Field notes were made during the interviews and focus groups. Why were field notes taken in relation to patient interviews at the patients' homes? What were they intended for? Were the interviews divided between WMC and IN, or did both participate in the interviews? How were the interviews conducted? Were the roles divided between who conducted the interview and who took field notes or..." |  | Line 165 | Field notes consisted of small notes on answers that stood out or interesting quotes. These could be used to make follow-up questions after a patients finished their answer. There were no notes on patients’ homes, just on their answers in the interviews. We changed field notes to “interview notes”.  Both WMC and IN participated in the interviews and took their own notes. WMC was generally more in the lead role during the interviews. | Line 180-181 |
| Did changes occur in the interview guide along the way? What were they? Why? |  | Line 166 | No big changes occurred. However we did sometimes ask participants to react to an answer of a previous interview. For example, one participant described test sessions as a facilitator. We asked other participants if they felt the same about test sessions.  We have added this to the text in “Data collection”. | Line 181-183 |
| In line 173 it is written ‘MvB and EG have previous experience with leading focus groups. But what is their professional background? |  | Line 170 | We added this in the text and also provided a reference to show MvB’s experience. | Line 190-191 |
| Who transcripted the interviews? |  | Line 181 | WMC, IN and FB transcripted the interviews. We added this in the text in “Data collection” | Line 179-180 |
| Which references was used regarding the inductive thematic analysis? |  | Line 182 | We have added the following references into the manuscript in data analysis  Michelle E. Kiger & Lara Varpio (2020) Thematic analysis of qualitative data: AMEE Guide No. 131, Medical Teacher, 42:8, 846-854, DOI: [10.1080/0142159X.2020.1755030](https://doi.org/10.1080/0142159X.2020.1755030)  Chapman AL, Hadfield M, Chapman CJ. Qualitative research in healthcare: an introduction to grounded theory using thematic analysis. J R Coll Physicians Edinb. 2015;45(3):201-5. doi: 10.4997/JRCPE.2015.305. PMID: 26517098. | Line 198-205 |
| You only describe how data saturation is achieved in patient interviews, what about in the focus groups? |  | Line 185 | We are uncertain if data saturation in the focus groups was achieved. We described this in the limitations section of the discussion. | Line 760-768 |
| The section on quality criteria for good qualitative research is good to include. However, for the manuscript to meet criteria regarding credibility, transferability, and dependability, a higher level of transparency is required regarding your methodological considerations and execution. |  | Line 191-206 | We believe that the methods section, thanks to your previous comments, is now more transparent. | - |
| In line 214 it is written ‘The median Clinical Frailty Score  was 3 (range 2-5)’. References? |  | Line 214 | We have added a reference for the Clinical Frailty score. | Line 231-232 |
| In line 220 you need to fill in the references |  | Line 220 | We could not find references that had to be filled in in line 220. | - |
| In qualitative research informations as mean age ect is not a result. The hole section from 208-220 need to be placed in the Method section. | Move to Methods section | Line 208-220, Line 222 | We appreciate your suggestion to move the description of participants including the demographic tables to the methods section. After careful consideration, with our research team, we believe that maintaining the current structure aligns better with the flow and coherence of our manuscript. | - |
| The same with Table 2 | Move to Methods section | Line 222 | See response to previous comment. | - |
| On what basis was it decided whether a factor was a facilitator or barrier? Was it based on a theory, model, or framework? Or something else? |  | Line 233 and Figure 2 | The presented factors are classified as facilitator or barrier based on how the participants portrayed it.  We have added a sentence in “results” to clarify. | Line 248-250 |
| The quote outlined in line 286 is somewhat leading. Considering that it is physiotherapists (health professionals) who are interviewing patients, it is worth considering whether it affects the patients' responses, especially with such a leading question. Perhaps the question is taken out of a broader context where initially more open-ended questions were asked, for example, if patients had any initiatives, they could mention that were significant to them? If such questions and data exist, I would replace the quote with a more open-ended question. |  | Line 286 | Good comment, we encountered while doing the interviews that patients did not have answers on those open questions. We started with open questions and if patients didn’t provide answers than we would check some of the suggestions we had. This specific question was in there because a part of our consortium was interested in what patients had to say to that question.  We have added a reflection on the background of the interviewers to the discussion. | Line 777-799 |
| In the results section line 305-351 more quotes need to be included to provide direct voices from participants and to support and illustrate the identified themes. Quotes add authenticity and depth to the results, contributing to the reader's understanding of participants' perspectives. | More quotes and interpretation of quotes | Line 305-351 | We have added three more quotes to the paragraph. | Lines 344-347, 360-365, 385-388 |
| The same applies to the section at line 368-399 | More quotes and interpretation of quotes. | Line 368-399 | We have added two more quotes to the paragraph | Lines 421-422 and 440-443 |
| The sub-theme 2.2 seems a bit thin and unsupported. Consider whether the content belongs in some of the other themes or expand on it. The finding that patients want a coordinator they don't have to deal with and repeat information to many health professionals a is an interesting result, which has been written about elsewhere. |  | Line 400-408 | There is indeed overlap between the themes as some factors are described in multiple themes. The research team debated whether we could fit 2.2. in the theme 2.3 but concluded that it should remain as a standalone theme as it is a unique part of the TFCP. We did change the name of 2.2 to fall assessment domain to be in accordance with the world guidelines for fall preventions and management in older adults. | Line 451 |
| Again, the citation in line 476 shows a quite close question. Think about if you have data on a more open question and replace. |  | Line 476 | We mainly used this quote to illustrate that patients themselves indicated that the PN might contact them. | - |
| In the results section, there are several instances where it is unclear who is speaking or interpreting what. Is it the research group, the patients, or the healthcare professionals, for example, at line 511-516. Or is it more of a discussion that belongs in the discussion section? Go through the results section and ensure that it is clear who is saying what, and if it is empirical data, support it with quotes. |  | Line 511-516 | We have scanned the results section for instances where it was unclear. In general, all factors were named by participants and not interpretations by the research team.  To clarify we have added this to the start of the results. | Line 249-250 |
| I am puzzled by the use of the term 'the shared decision-making conversation' throughout the manuscript. Shared decision-making is a standardized process that often requires training and is sometimes supported by a decision support tool. I do not understand how the term is used here, and there are no references. |  | e.g. line 552 | With shared decision making conversation, we refer to the conversation between the patient and healthcare professional during the shared decision making process.  We adjusted this throughout the manuscript by removing “conversation” for clarification and added a reference.  Elwyn G, Frosch D, Thomson R, Joseph-Williams N, Lloyd A, Kinnersley P, Cording E, Tomson D, Dodd C, Rollnick S, Edwards A, Barry M. Shared decision making: a model for clinical practice. J Gen Intern Med. 2012 Oct;27(10):1361-7. doi: 10.1007/s11606-012-2077-6. Epub 2012 May 23. PMID: 22618581; PMCID: PMC3445676. | Line 149-151, 605 |
| Could you explain the difference described in line 660-661? |  | Line 660-661 | Patients in our study wanted the intervention to be tailored to their “level”. So for example, they did not want to start with hard balance exercises if they were not ready for it. In the literature study, patients experienced health related issues, such as chest and joint pain, that would limit their participation.  We elaborated this in the new version. | Line 714-716 |
| Again, do you really mean to develop new implementation strategies? Many of the proposed strategies you have mentioned so far are described in articles from implementation research literature. I am missing a definition of what you understand by implementation strategies, starting from the first time you mention it in the manuscript. |  | Line 669-670 | The aim of this study was to identify facilitators and barriers. Based on these factors, we will choose implementation strategies. Using the present barriers and facilitators we will fill out the CFIR ERIC Tool from the Consolidated Framework for Implementation Research Team to derive the implementation strategies.  We use the following definition of implementation strategy: “Implementation strategies can be defined as methods or techniques used to enhance the adoption, implementation, and sustainability of a clinical program or practice.”  Proctor EK, Powell BJ, McMillen JC. Implementation strategies: recommendations for specifying and reporting. Implement Sci. 2013 Dec 1;8:139. doi: 10.1186/1748-5908-8-139. PMID: 24289295; PMCID: PMC3882890.  We have added this definition and source to the first time we mention implementation strategy in the background. | Line 82-86 |
| In consideration that it is a thematic content analysis allowing you to delve deeper than the spoken words, I do not perceive a discussion that goes particularly deep. It becomes more of a description of which barriers and facilitators are similar to other studies than an elaboration. For example, it's nice to find that patients have preferences for coordination or communication between entities. However, critically addressing how challenging it is to get entities to communicate at all could be explored more. Many of your results could be discussed more in-depth. | e.g. references: It is two worlds” cross-sectoral nurse collaboration related to care transitions: A qualitative study  Helle Vendel Petersen, Signe Foged, Vibeke Nørholm  Allen, J., Ottmann, G., & Roberts, G. (2013). Multi‐professional communication for older people in transitional care: A review of the literature.  International Journal of Older People Nursing,  Axelsson, S. B., & Axelsson, R. (2009). From territoriality to altruism in interprofessional collaboration and leadership.  Journal of Interprofessional Care, 23(4), 320–330. https://doi.  org/10.1080/13561820902921811  Bauer, M., Fitzgerald, L., Haesler, E., & Manfrin, M. (2009). Hospital  discharge planning for frail older people and their family.  Are we delivering best practice? A review of the evidence.  Journal of Clinical Nursing, 18(18), 2539–2546. https://doi.  org/10.1111/j.1365-2702.2008.02685.x |  | We have elaborated our discussion with a more in depth continuation on interprofessional collaboration, high staff turnover in the ED, and the reality of implementation. Additionally, we included a paragraph on the influence of a constructivist paradigm and the lack of framework in the development of the interview guide. | Line 731-754 |
| In terms of strengths and weaknesses, I miss a discussion on the use of a constructivist paradigm in this study. What has it contributed? How has it sharpened your analysis and discussion? Pros and cons of being an insider versus an outsider when conducting qualitative studies, including being a therapist with knowledge of the object? Strengths and weaknesses of not working with theories, models, and frameworks when the focus (also) is on how to implement a new intervention? What does the literature say about this? |  | Line 677-698 | We have added the following text to the strengths and limitations:  We believe that the use of a constructivist paradigm contributed to our results as it emphasizes the subjective nature of perceived realities and the differences in individuals’ perspectives. By exploring these multiple perspectives, we could provide a more comprehensive and inclusive understanding of multiple themes. The interaction between researchers and participants influenced the results due to the radical subjectivism. The background of the interviewers could in this case impact the results as it might have pushed it more towards the perspectives of healthcare professionals rather than patient perspectives. By pilot testing the interviews with patient representatives we did our best to prevent this bias from happening. The background of the interviewers did also contribute to more specific follow-up questioning about for example patients’ experiences with treatment. As the interviewers were active in patient care they were more familiar with barriers or facilitators that could arise during treatment. Moreover, by having knowledge on the TFCP’s steps, the interviewers were able to explain the steps to the patients, ensuring higher quality of discussions.  In implementation research, interview guides are often shaped by a conceptual framework such as the Consolidated Framework for Implementation Research (CFIR) (1, 2). In our study, we used the concept of the TFCP as a conceptual basis in development of our interview guide. Instead of using the CFIR we wanted a to hear from the participants themselves what was most important to them while incorporating some constructs of the CFIR such as “adaptability”, “available resources” and “access to knowledge & information”. In hindsight, the results would have been different if we shaped our interview guide based on the CFIR constructs. As these constructs are general more focussed on corporate structures or opportunities and less on personal factors. Our current approach ensured a strong personal voice for patients and healthcare professionals. | Line 777-799 |
| Think about the sentence ‘to design context-tailored implementation strategies.’ Context-tailored is not defined as a concept! |  |  | We have removed “context” from the sentence as “tailored” is already clear enough. | Line 803-804 |
| Several revisions are needed for the manuscript to be recommended for publication. These mainly concern transparency so that the study can meet the criteria for good qualitative research. |  |  | We hope our manuscript with revisions can be recommended for publication. |  |
