# Supplementary material for: Facilitators and barriers for the implementation of a transmural fall-prevention care pathway for older adults in the emergency department
Source: PLoS One. 2024 Dec 31;19(12):e0314855. doi: 10.1371/journal.pone.0314855 (PMC11687785; doi:10.1371/journal.pone.0314855)
Supplement: Attachment — Submitted filename: Response to reviewers November 2024.docx [file pone.0314855.s010.docx]

Dear Editor,

We would like to thank you for the dedicated effort to secure reviewers for our manuscript “Facilitators and barriers for the implementation of a transmural fall-prevention care pathway for older adults in the emergency department”.

We are grateful for the acceptance by reviewer 1 and the constructive feedback from reviewer 3. We have considered the revisions and made adjustments in the manuscript based on the reviewers’ suggestions. Please see below our responses to the reviewers’ questions and comments on a point by point basis.

Kind regards, on behalf of all co-authors,

Wilmar Charmant, MSc

**Reviewer 3 comments to the manuscript:** Facilitators and barriers for the implementation of a transmural fall-prevention care pathway for older adults in the emergency department.

**Manuscript** #: PONE-D-23-33190

**Title:** Facilitators and barriers for the implementation of a transmural fall-prevention care pathway for older adults in the emergency department

**Authors**: Willem Maarten Charmant; Barbara Snoeker; Hein van Hout; Ilse Nauta; Femke Boonstra; Edwin Geleijn; Cindy Veenhof; Prabath Nanayakkara

**Article type:** Research Article

| Comments | Line | Author’s response | Adjusted lines in the unmarked version |
| --- | --- | --- | --- |
| Thank you very much for allowing me to review your manuscript. Congratulations on your work, and I am sending some minor revisions that I hope will further enhance the message you wish to convey to readers.  I hope my recommendations will help improve the article's clarity and precision and strengthen the practical applicability of the findings for other researchers and professionals in the field of fall prevention among older adults in hospital settings. |  | Thank you for your compliments regarding our work. We believe that we further improved our manuscript with your feedback. |  |
| While the sample size is mentioned, it would be beneficial to clarify why ten older adults and thirteen healthcare professionals were selected. Expanding the description of participant diversity, such as demographic and professional backgrounds, could enrich the study's applicability. | Methods | The number of participants was not predetermined but related to data saturation as mentioned in lines 109-110. Since 9 to 17 interviews are common qualitative studies, we aimed to begin within this range and assessed data saturation throughout the process ^1^.  We agree that including more detailed demographic characteristics could have added value to this study. Unfortunately, information such as ethnicity, educational status, or income was not gathered during the study and was not consistently available in the electronic patient files. Additionally, some of these variables are sensitive and require careful consideration when collecting them. Meanwhile, a number of participants have sadly passed away, limiting our ability to retrieve this information retrospectively.   1. Monique Hennink, Bonnie N. Kaiser, Sample sizes for saturation in qualitative research: A systematic review of empirical tests, Social Science & Medicine, Volume 292, 2022, 114523, ISSN 0277-9536, <https://doi.org/10.1016/j.socscimed.2021.114523>. (https://www.sciencedirect.com/science/article/pii/S0277953621008558) | NA |
| Regarding the interviews conducted in patients' homes, the description of the field notes could be more specific. Providing additional details on how these notes were used and the basis for their utility would be helpful. | Methods | We elaborated on the notes by adding a sentence in the methods.  *During the interviews and focus groups, WMC, IN, and FB documented field notes to support the flow of the conversation by marking points to revisit as needed and recording notable insights. These notes also served as a reference for observations made during the discussions, allowing verification of whether these themes emerged in the subsequent data coding process.* | 181-185 |
| The text acknowledges that data saturation was not reached in the focus groups, representing a limitation in interpreting results. It also mentions that workload and staffing changes restricted the participation of some relevant professionals. Emphasizing how this limitation affects the generalizability of the findings would be valuable.  Considering that the study was conducted in an area with a high socioeconomic level, it is suggested that facilitators and barriers may vary in settings with different socioeconomic conditions. Indicating how this limits the findings' applicability to other contexts could strengthen the limitations section. | Discussion | We have improved the limitations sections to further clarify the effect of the mentioned limitations on the generalisability of our findings.  *Older adults in the VUmc area generally have a relatively high socioeconomic status, which may influence the generalisability of the found facilitators and barriers. In lower socioeconomic areas, differing healthcare access might reveal additional or more prominent barriers that were less emphasized in this study (52). Additionally, data saturation with the healthcare professionals may not have been reached. Due to the high workload in primary care during the COVID pandemic, we were unable to organise a focus group with all relevant healthcare professionals at the same time. The high workload also caused certain professions, such as the GP, to be underrepresented in our study. This may impact the generalisability of the results as the perspectives of a few individuals may not fully represent the entire professional group.*  52. McMaughan DJ, Oloruntoba O, Smith ML. Socioeconomic Status and Access to Healthcare: Interrelated Drivers for Healthy Aging. Front Public Health. 2020 Jun 18;8:231. doi: 10.3389/fpubh.2020.00231. PMID: 32626678; PMCID: PMC7314918. | 759-768 |
|  | Discussion |  |  |
| The current conclusion provides a good overview, but reinforcing the recommendation to implement intervention strategies in contexts with similar characteristics would be useful. | Conclusion | We have improved to conclusion by implementing your suggestion:  It is crucial to consider facilitators and barriers from the perspectives of both patients and healthcare professionals for the implementation of a TFCP in communication, organisation & execution, and personal factors. The results of this study provide useful information to design tailored implementation strategies. The implementation of a TFCP will guide patients towards fall preventive interventions, ultimately reducing the risk of recurrent falls and fall-related ED visits. *The found themes are relevant to take into account when designing implementation strategies in all settings, but future research is needed to explore how additional or different barriers may emerge in lower socioeconomic areas.* Furthermore, future research should evaluate the effectiveness of tailored implementation strategies for the implementation of a TFCP. | 806-808 |
