# Supplementary material for: Facilitators and barriers for the implementation of a transmural fall-prevention care pathway for older adults in the emergency department
Source: PLoS One. 2024 Dec 31;19(12):e0314855. doi: 10.1371/journal.pone.0314855 (PMC11687785; doi:10.1371/journal.pone.0314855)
Supplement: Attachment — Submitted filename: Reviewer comments to the manuscript.docx [file pone.0314855.s008.docx]

**Reviewer comments to the manuscript:** Facilitators and barriers for the implementation of a transmural fall-prevention care pathway for older adults in the emergency department.

**Manuscript** #: PONE-D-23-33190

**Title:** Facilitators and barriers for the implementation of a transmural fall-prevention care pathway for older adults in the emergency department

**Authors**: Willem Maarten Charmant; Barbara Snoeker; Hein van Hout; Ilse Nauta; Femke Boonstra; Edwin Geleijn; Cindy Veenhof; Prabath Nanayakkara

**Article type:** Research Article

| Comments | Possible changes | Line |
| --- | --- | --- |
| Thank you for the opportunity to review this manuscript. It focuses on a highly clinically relevant issue, namely, fall patients. In terms of the manuscript's quality regarding qualitative studies, several revisions are needed. See comments below. |  |  |
| On line 81, it is written, 'to develop implementation strategies,' but often, we choose existing described strategies, as seen in, for example, Powell et al., 2012, 2015, rather than developing our own implementation strategies. | So, perhaps it is more accurate to write 'to select and/or develop...' | Line 81 |
| The section on the epistemological standpoint lacks proper references. |  | Line 97-101 |
| In line 121 is written ‘The healthcare professionals were  122 familiar with care for older adults after a fall’. What does familiar means in the context of recruitment of health care professionals?  Were other selected inclusion criteria used, such as general professional experience and experience with the specialty? Why or why not?" |  | Line 121-122 |
| Could you elaborate a bit more on the choices you made regarding settings? Why were interviews conducted in the patients' homes and not at the hospital? Why did you choose to categorize healthcare professionals into primary and secondary staff when your objective was to develop a cross-sectoral pathway? |  | Line 126-133 |
| On line 136, it is written ‘We designed the concept of TFCP for older adults after a fall-related ED visit’. I wonder who 'we' are. Is it the research group behind the study? What is the basis for developing this program? Is it based on experiences, evidence-based literature, inputs from users etc.? How was this intervention developed?" |  | Line 236-145 |
| In line 152 it is mentioned ‘were asked for their demographic characteristics’. It's a very broad and generalized statement. Could you elaborate on which demographic data and why these? |  | Line 152 |
| Line 155 was the interview guide develop in collaboration with the entire research group or selected researchers. Who and why? |  | Line 155 |
| On line 156 it is written ‘After and during the discussion of  157 these themes, we reserved time for new input’. What does ‘we reserved time for new input’ means? Who was supposed to provide new inputs? When? Was it part of a pilot test of your interview guide or...? |  | Line 156 |
| Why are WMC and IN conducting individual interviews? Who are they? Similarly, why are WMC, FB, MvB, and EG conducting the focus group interviews? Did all four participate in each focus group interview, or were they divided? How many patients in total participated, and how was this number chosen? The same goes for healthcare professionals? |  | Line 159-162 |
| "Why did you choose focus group interviews instead of group interviews? What was your purpose with the focus group interviews? Which references did you use regarding interviews and focus group interviews? |  |  |
| Was the interview guide outlined in the table used for individual interviews with the patients? It seems that several of the themes would be challenging for a patient to respond to. What did the interview guide look like for the patients? |  | Table 1 |
| In line 165, it is written: Field notes were made during the interviews and focus groups. Why were field notes taken in relation to patient interviews at the patients' homes? What were they intended for? Were the interviews divided between WMC and IN, or did both participate in the interviews? How were the interviews conducted? Were the roles divided between who conducted the interview and who took field notes or..." |  | Line 165 |
| Did changes occur in the interview guide along the way? What were they? Why? |  | Line 166 |
| In line 173 it is written ‘MvB and EG have previous experience with leading focus groups. But what is their professional background? |  | Line 170 |
| Who transcripted the interviews? |  | Line 181 |
| Which references was used regarding the inductive thematic analysis? |  | Line 182 |
| You only describe how data saturation is achieved in patient interviews, what about in the focus groups? |  | Line 185 |
| The section on quality criteria for good qualitative research is good to include. However, for the manuscript to meet criteria regarding credibility, transferability, and dependability, a higher level of transparency is required regarding your methodological considerations and execution. |  | Line 191-206 |
| In line 214 it is written ‘The median Clinical Frailty Score  was 3 (range 2-5)’. References? |  | Line 214 |
| In line 220 you need to fill in the references |  | Line 220 |
| In qualitative research informations as mean age ect is not a result. The hole section from 208-220 need to be placed in the Method section. | Move to Methods section | Line 208-220 |
| The same with Table 2 | Move to Methods section | Line 222 |
| On what basis was it decided whether a factor was a facilitator or barrier? Was it based on a theory, model, or framework? Or something else? |  | Line 233 and Figure 2 |
| The quote outlined in line 286 is somewhat leading. Considering that it is physiotherapists (health professionals) who are interviewing patients, it is worth considering whether it affects the patients' responses, especially with such a leading question. Perhaps the question is taken out of a broader context where initially more open-ended questions were asked, for example, if patients had any initiatives, they could mention that were significant to them? If such questions and data exist, I would replace the quote with a more open-ended question. |  | Line 286 |
| In the results section line 305-351 more quotes need to be included to provide direct voices from participants and to support and illustrate the identified themes. Quotes add authenticity and depth to the results, contributing to the reader's understanding of participants' perspectives. | More quotes and interpretation of quotes | Line 305-351 |
| The same applies to the section at line 368-399 | More quotes and interpretation of quotes. | Line 368-399 |
| The sub-theme 2.2 seems a bit thin and unsupported. Consider whether the content belongs in some of the other themes or expand on it. The finding that patients want a coordinator they don't have to deal with and repeat information to many health professionals a is an interesting result, which has been written about elsewhere. |  | Line 400-408 |
| Again, the citation in line 476 shows a quite close question. Think about if you have data on a more open question and replace. |  | Line 476 |
| In the results section, there are several instances where it is unclear who is speaking or interpreting what. Is it the research group, the patients, or the healthcare professionals, for example, at line 511-516. Or is it more of a discussion that belongs in the discussion section? Go through the results section and ensure that it is clear who is saying what, and if it is empirical data, support it with quotes. |  | Line 511-516 |
| I am puzzled by the use of the term 'the shared decision-making conversation' throughout the manuscript. Shared decision-making is a standardized process that often requires training and is sometimes supported by a decision support tool. I do not understand how the term is used here, and there are no references. |  | e.g. line 552 |
| Could you explain the difference described in line 660-661? |  | Line 660-661 |
| Again, do you really mean to develop new implementation strategies? Many of the proposed strategies you have mentioned so far are described in articles from implementation research literature. I am missing a definition of what you understand by implementation strategies, starting from the first time you mention it in the manuscript. |  | Line 669-670 |
| In consideration that it is a thematic content analysis allowing you to delve deeper than the spoken words, I do not perceive a discussion that goes particularly deep. It becomes more of a description of which barriers and facilitators are similar to other studies than an elaboration. For example, it's nice to find that patients have preferences for coordination or communication between entities. However, critically addressing how challenging it is to get entities to communicate at all could be explored more. Many of your results could be discussed more in-depth. | e.g. references: It is two worlds” cross-sectoral nurse collaboration related to care transitions: A qualitative study  Helle Vendel Petersen, Signe Foged, Vibeke Nørholm  Allen, J., Ottmann, G., & Roberts, G. (2013). Multi‐professional communication for older people in transitional care: A review of the literature.  International Journal of Older People Nursing,  Axelsson, S. B., & Axelsson, R. (2009). From territoriality to altruism in interprofessional collaboration and leadership.  Journal of Interprofessional Care, 23(4), 320–330. https://doi.  org/10.1080/13561820902921811  Bauer, M., Fitzgerald, L., Haesler, E., & Manfrin, M. (2009). Hospital  discharge planning for frail older people and their family.  Are we delivering best practice? A review of the evidence.  Journal of Clinical Nursing, 18(18), 2539–2546. https://doi.  org/10.1111/j.1365-2702.2008.02685.x |  |
| In terms of strengths and weaknesses, I miss a discussion on the use of a constructivist paradigm in this study. What has it contributed? How has it sharpened your analysis and discussion? Pros and cons of being an insider versus an outsider when conducting qualitative studies, including being a therapist with knowledge of the object? Strengths and weaknesses of not working with theories, models, and frameworks when the focus (also) is on how to implement a new intervention? What does the literature say about this? |  | Line 677-698 |
| Think about the sentence ‘to design context-tailored implementation strategies.’ Context-tailored is not defined as a concept! |  |  |
| Several revisions are needed for the manuscript to be recommended for publication. These mainly concern transparency so that the study can meet the criteria for good qualitative research. |  |  |
